# A case report of sudden cardiac arrest and torsade de pointes induced by the second-generation tyrosine kinase inhibitor dasatinib combined with fluconazole

**DOI:** 10.3389/fcvm.2023.984572

**Published:** 2023-02-15

**Authors:** Yuting Yuan, Chunjian Wang, Hongying Yao

**Affiliations:** ^1^Department of Cardiology, Peking University International Hospital, Beijing, China; ^2^Department of Hematology, Peking University International Hospital, Beijing, China

**Keywords:** dasatinib, fluconazole, drug-induced long QT syndrome, torsade de pointes, tyrosine kinase inhibitor, sudden cardiac arrest, case report

## Abstract

A-41-year-old man diagnosed with acute myeloid leukemia (AML) survived dasatinib + fluconazole drug-induced long QT syndrome, sudden cardiac arrest, and torsade de pointes. Drug features and interaction jointly contributed to the whole process. Therefore, appropriate attention to drug interaction and close ECG monitoring are highly recommended for hospitalized patients, especially for those undergoing multi-drug regimens.

## Introduction

Cardiac arrhythmia is an emerging and insufficiently recognized concern of anticancer drugs, resulting mostly from the use of an increasing number of targeted therapies, such as tyrosine kinase inhibitors (TKI) (1). In a World Health Organization pharmacovigilance study, 40 anticancer drugs, which were mostly kinase inhibitors (41%), were significantly associated with drug-induced long QT syndrome, which may deteriorate into morphologically distinctive polymorphic ventricular arrhythmia, torsade de pointes (Tdp), and sudden cardiac death (2). In this report, we present a patient with acute myeloid leukemia (AML) who, during hospitalization, survived Tdp and sudden cardiac arrest, which were caused by multiple factors, such as dasatinib and fluconazole co-administration and patient factors.

## Case presentation

### Timeline

**Table 1:** 

Time point	Event
September 2021	Diagnosed with AML and treated with induction therapy (HAA)
October 2021	Diagnosed with invasive fungal disease and administered amphotericin B formulation and caspofungin acetate therapy
January 2022	Administered round 1 VEN + AZA
February 2022	Liver biopsy revealed *Candida* tropicalis infection
March 2022	Administered round 2 VEN + AZA
April 2022	Hospitalized due to fungal infection; antifungals, including micafungin, fluconazole, and antibacterial ceftazidime, were administered
Day 24 in hospital	100 mg dasatinib was administered orally
Day 25 in hospital	Sudden cardiac arrest and torsade de pointes occurred
Day 28 in hospital	Torsade de pointes and QTc resolved
Day 32 in hospital	Discharged from hospital
July 2022	Underwent chemotherapy in another hospital during follow-up

A 41-year-old man with a 6-month history of AML was admitted to our hospital for an invasive fungal infection of the lung, liver, and blood of a 4-month duration. In September 2021, the patient first presented to another hospital with fatigue and gingival bleeding. His complete blood count (CBC) was as follows: white blood cell, 4.48 × 10^9^/L; hemoglobin, 71 g/dL; and platelets, 21 × 10^9^/L. Bone marrow aspiration revealed the presence of blast cells (60%). Flow cytometry revealed 35% abnormal myeloid blasts expressing CD34, CD38, CD117, HLA-DR, CD13, CD33, and MPOdim. These abnormal myeloid blasts partially expressed CD19, CD15, and CD71, but lacked CD5, CD7, CD10, and CD14. Karyotype analysis showed 45,X,-Y,t(8,21)(q22;q22) in 20 analyzed metaphases. Next-generation sequencing revealed the presence of KIT (p.N822K). The diagnosis of AML [M2, ETO+, c-KIT (N 822+)] was made. Thereafter, the patient underwent induction chemotherapy, consisting of aclarubicin, homoharringtonine, and cytarabine. Then, 15 days later, the patient experienced diarrhea, fever, and pneumonia. The test results were suggestive of invasive fungal disease. Antifungal agents, including amphotericin B formulation and caspofungin acetate, were administered. In January and March 2022, the patient underwent two rounds of venetoclax (VEN) and azacitidine (AZA) chemotherapies separately. However, the infection was not thoroughly controlled; it gradually invaded the liver in the following months. Next-generation sequencing of a liver biopsy obtained in February 2022 confirmed *Candida* tropicalis infection. Thereafter, he was admitted to our hospital in April 2022. His medical and family histories were unremarkable.

**Figure 1:**
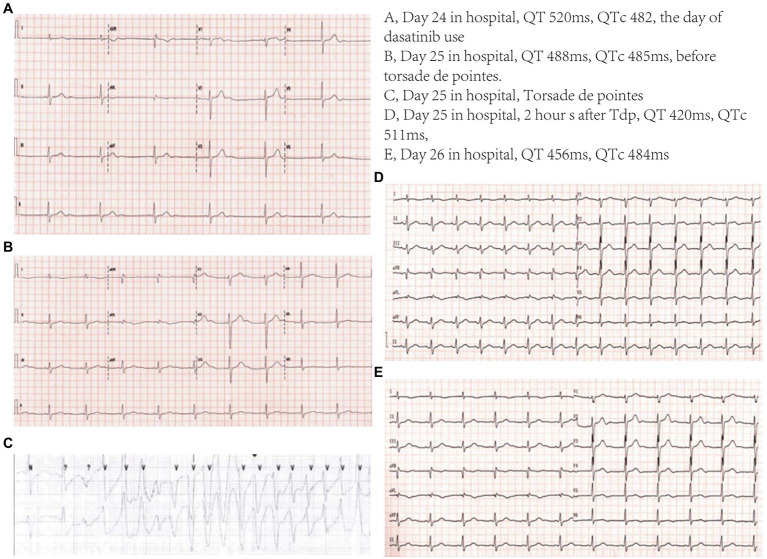
Electrocardiogram.

**Figure 2:**
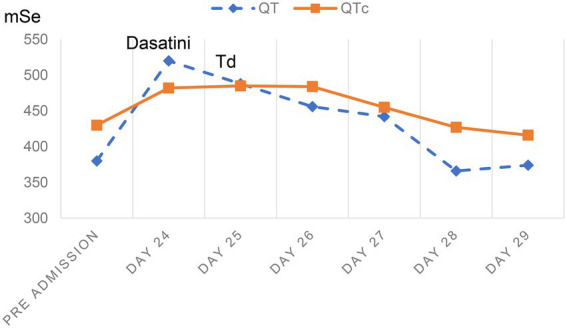
QT and QTc changes.

On admission, echocardiography and electrocardiography findings were unremarkable. Antifungals, including micafungin, fluconazole, and antibacterial ceftazidime, were administered intravenously. Approximately 24 days after admission, 100 mg of dasatinib was administered orally. Thereafter, the patient complained of palpitations. Blood pressure was markedly elevated to 204/98 mmHg. The temperature, heart rate, and respiratory rate were 36.5°C, 42 beats per minute, and 15 breaths per minute, respectively. Electrocardiography showed remarkable sinus bradycardia, heart rate of 38 beats per minute, QT interval of 520 ms, and QT correction (QTc) interval of 482 ms (Figure 1). No ST segment deviation and T wave changes were observed. The complete blood count (CBC) was as follows: white blood cell, 7.53 × 10^9^/L; hemoglobin, 115 g/dL; and platelets, 96 × 10^9^/L. His full blood chemistry panel showed slight hypokalemia (3.22 mmol/L, reference level 3.5–5.5 mmol/L) and normal serum creatine and liver enzyme levels. NT-proBNP concentration was 434 pg./mL (reference level is ≤125 pg./mL), and the hyper-sensitive cardiac troponin T (hs-cTnT) level was 22.8 ng/L (reference level < 14 ng/L). The plasma magnesium level was 0.86 mmol/L (reference level = 0.75–1.02 mmol/L). Atropine was administered to increase the heart rate, whereas sodium nitroprusside was administered to decrease the blood pressure. Additionally, oral and intravenous potassium supplements were administered. On the second day, the potassium level increased to 3.68 mmol/L. At 1 p.m., the patient suddenly lost consciousness, and cardiopulmonary resuscitation was immediately initiated. The electrocardiography on the defibrillator showed ventricular fibrillation, and electrical cardioversion was performed simultaneously. The patient regained full consciousness and was transferred to the cardiac intensive care unit. Several episodes of torsade de pointes (Figure 1) were detected on cardiac telemetry monitoring; however, these episodes did not affect hemodynamic stability. Fluconazole and dasatinib were suspected to have induced these hemodynamic changes and were discontinued. Thereafter, intravenous isoproterenol was administered to elevate the heart rate to >90 beats per minute and shorten the QT interval. Aggressive potassium supplements were used to increase the potassium level to >4 mmol/L. Magnesium supplements, including magnesium sulfate and potassium magnesium aspartate, were added. The vicious arrhythmia abated, and the QTc interval gradually returned to 427 ms (QT interval returned to 366 ms) in the 3 days following the event. The longitudinal QT change is shown in Figure 2. Then, 5 days later, hs-cTnT and NT-proBNP levels were 23.4 ng/L and 162.1 pg./mL, respectively. Echocardiography revealed consistent normal left ventricular function. The patient was discharged after prolonged monitoring and duly educated about the prohibited use of certain drugs. Then, 2 months later during follow-up, the patient underwent chemotherapy in another hospital.

## Discussion

The development of various anti-cancer therapeutics has improved the prognosis and increased the survival time of patients with different malignancies (3). However, associated cardiovascular adverse drug reactions, such as heart failure (4), acute myocardial infarction (5), myocarditis (6), vicious arrhythmia, and sudden cardiac death (1), have been the concern of clinicians. A drug-induced long-QT syndrome is an important adverse reaction of anti-tumor drugs, which can further deteriorate into Tdp, ventricular fibrillation, and sudden cardiac death. QTc prolongation refers to corrected QT prolongation > 450 ms in men and >470 ms in women (7) or an absolute increase of >60 ms above baseline. The risk of Tdp increases by 5%–7% for every 10-ms extension in the QTc interval (8), and QTc > 500 ms is considered to be remarkably associated with Tdp (9). However, the relationship between the changes in QT and Tdp widely varies. Tdp may occur when QTc is mildly prolongated, whereas, in some individuals, no events result from markedly prolonged QT (10). In this case, QTc was slightly elevated, which triggered Tdp and ventricular fibrillation. Herein, QT prolongation was not the cause of Tdp, but the manifestation of the increase in dispersion of repolarization, which provided the arrhythmogenic substrate and resulted in Tdp (11). Transmural dispersion of repolarization has been proven to be an indicator of this abnormality (12).

Dasatinib, the second-generation tyrosine kinase inhibitor, has been reported to suppress various types of kinases, including BCR-ABL1, FGFR2, c-KIT, PDGFRα, PDGFRβ, EPHA, and Src family (13, 14). Therefore, the drug is now being used to treat patients with leukemia having targeted chromosomal changes. This patient went through recurrent fungal and bacterial infections, which restricted the periodic administration of chemotherapy. After admission, his response to antifungal therapy was poor, and bone marrow aspiration revealed a possible AML relapse. Considering the role of c-KIT mutation in AML and the use of dasatinib, according to the NCCN 2022 guideline, in this kind of patient (15, 16), the hematologist decided to administer 100 mg of dasatinib, instead of chemotherapy, to target the c-KIT mutation. Dasatinib is rapidly absorbed, and at least 80% of the oral dose is deemed to be bioavailable. Dasatinib is eliminated through CYP3A4-mediated metabolism, with a relatively short half-life of approximately 5–6 h. Dasatinib pharmacokinetics are not influenced by age, race, and renal insufficiency. Clinicians need to be cautious of potential drug interactions between dasatinib and pH-modifying agents (17) and strong inducers/inhibitors of CYP3A (18).

According to the data extracted from adverse event reports recorded in the publicly available version of the United States Food and Drug Administration (FDA) Adverse Event Reporting System (FAERS) database, dasatinib can induce torsade de pointes/QT prolongation (at a relatively low rate) and pulmonary hypertension (19). Spechbach et al. (20) presented a case of dasatinib-induced reversible ventricular arrhythmia. Considering its pharmacokinetic characteristics, dasatinib is mostly metabolized by cytochrome P450 3A4 (CYP3A4). Hence, it is subjected to triazoles, and its risk of toxicity and half-life can be largely augmented when exposed simultaneously (18). Another tyrosine kinase inhibitor, osimertinib, when combined with *litsea cubeba*, has been reported to cause Tdp (21). Monitoring the plasma concentration of dasatinib may be helpful to determine the safe range. The safe concentration of dasatinib not causing pleural effusion has been determined (22), but data about Tdp are lacking. Unfortunately, we are unable to determine TKI plasma concentrations in our hospital, so were unable to establish if dasatinib plasma concentration was elevated at the time when the patient developed TdP. Ongoing measurement of dasatinib plasma levels may have allowed the patient to safely continue dasatinib treatment following either a dose reduction or cessation of the interacting drugs.

Fluconazole is a triazole antifungal agent that inhibits the growth of fungi by inhibiting ergosterol production. It is commonly used to treat opportunistic fungal infections caused by *Candida*, *Cryptococcus*, and other fungal species. It has been proven to prolong the QT interval and endocardial ventricular action potential duration, increase transmural dispersion of repolarization, and induce early afterdepolarizations (EADs) in rabbit models (23). It was found to induce torsade de pointes in children (24) and adults, when administered singly or combined with other drugs, such as amitriptyline (25), fluoroquinolone (26), and arsenic trioxide (27). Acting as a potent inhibitor of cytochrome P450 3A4 (CYP3A4), fluconazole, may exhibit indefinite clinical effects due to drug interactions. In our case, the patient responded to fluconazole treatment only in the first 24 days but experienced sudden cardiac arrest when dasatinib was added. Herein, after excluding other possible factors, we suspected the reactions to be due to the drug features of dasatinib and its drug interactions with fluconazole.

Several risk factors contribute to the development of Tdp in hospitalized patients; they include QTc > 500 ms, QT-prolonging drug use, heart disease (congestive heart failure and myocardial infarction), advanced age, sex (woman), electrolyte disturbance (hypokalemia, hypomagnesemia, hypocalcemia, etc.), diuretic treatment, impaired hepatic drug metabolism (hepatic dysfunction or drug–drug interactions), bradycardia, and genetic polymorphisms (28). Particularly, drug interactions are easily neglected in clinical practice. Meanwhile, in our case, extracellular potassium increased, but intracellular potassium was unclear and potassium imbalance should be considered as one factor predisposing to arrhythmia. In clinical practice, QTc prolongation may occur without deteriorating into Tdp, as occurs with amiodarone use. Transmural dispersion of repolarization seems important but still lacks valid markers for monitoring and needs further investigation.

## Conclusion

In this report, a 41-year-old man experienced sudden cardiac arrest and Tdp after dasatinib and fluconazole co-administration. Based on this case, clinicians should be cautious of the potential cardiac side effects of dasatinib and the possible drug interaction between dasatinib and CYP3A4 inhibitors. Additionally, the case accentuates the necessity for ECG monitoring during anticancer drug administration.

## Ethics statement

Ethical review and approval was not required for the study on human participants in accordance with the local legislation and institutional requirements. The patients/participants provided their written informed consent to participate in this study and for the publication of this case report.

## Author contributions

YY contributed to manuscript writing and data collection. CW and HY contributed to clinical management. All authors contributed to the article and approved the submitted version.

## Funding

This study was funded by the Peking University International Hospital.

## Conflict of interest

The authors declare that the research was conducted in the absence of any commercial or financial relationships that could be construed as a potential conflict of interest.

## Publisher’s note

All claims expressed in this article are solely those of the authors and do not necessarily represent those of their affiliated organizations, or those of the publisher, the editors and the reviewers. Any product that may be evaluated in this article, or claim that may be made by its manufacturer, is not guaranteed or endorsed by the publisher.
